# Draft Genome Sequence of *Aliiroseovarius crassostreae* CV919-312, the Causative Agent of *Roseovarius* Oyster Disease (Formerly Juvenile Oyster Disease)

**DOI:** 10.1128/genomeA.00148-16

**Published:** 2016-03-17

**Authors:** Linda Kessner, Edward Spinard, Marta Gomez-Chiarri, David C. Rowley, David R. Nelson

**Affiliations:** a Department of Cell and Molecular Biology, University of Rhode Island, Kingston, Rhode Island, USA; b Department of Fisheries, Animal and Veterinary Sciences, University of Rhode Island, Kingston, Rhode Island, USA; c Department of Biomedical and Pharmaceutical Sciences, University of Rhode Island, Kingston, Rhode Island, USA

## Abstract

*Aliiroseovarius crassostreae* CV919-312 is a marine alphaproteobacterium and the causative agent of *Roseovarius* oyster disease. We announce here the draft genome sequence of *A. crassostreae* CV919-312 and identify potential virulence genes involved in pathogenicity.

## GENOME ANNOUNCEMENT

*Aliiroseovarius crassostreae*, formerly known as *Roseovarius crassostreae*, is the causative agent of *Roseovarius* oyster disease (ROD), which results in high mortality rates in hatchery-raised juvenile eastern oysters in the northeast United States (1–3). This alphaproteobacterium is a strict aerobe and a member of the *Roseobacter* clade (4). Strain CV919-312 was isolated by Boettcher et al. (4) from tissues of an infected oyster from the Damariscotta River in Maine. Here, we announce the genome sequence of *A. crassostreae* CV919-312Sm, a spontaneous streptomycin-resistant mutant, to help elucidate the mechanisms of virulence used by this bacterium to cause ROD.

A single isolated colony of *A. crassostreae* CV919-312Sm was grown overnight in yeast-peptone broth supplemented with 3% artificial sea salts (YP30) (5). Genomic DNA was isolated using the Promega Wizard genomic DNA purification kit, and DNA was resuspended in 2 mM Tris-HCl buffer (Bio Basic). Sequencing was performed at the Rhode Island Genomics and Sequencing Center using the Illumina MiSeq benchtop instrument.

Sequence trimming and *de novo* assembly were performed using the CLC Genomics Workbench (version 8.0.1). Contigs with an average coverage of >100× were assembled with the CLC Genomics Workbench and SPAdes genomic assembler (version 3.1.1). The resulting contigs were joined using the CLC Microbial Genome Finishing module. The draft genome consists of 26 contigs, with a total sequence length of 3,706,831 bp and G+C content of 58.4%, plus one complete plasmid of 18,548 bp with a G+C content of 58.3%.

Gene annotation was performed using Rapid Annotations using Subsystems Technology (RAST) (6–8) and Integrated Microbial Genomes/Expert Review (IMG/ER) (9). Annotation revealed multiple clusters of genes involved in *flp* (fimbrial low-molecular-weight) pilus assembly, which may play an important role in attachment to the inner shell surface of oysters. Oyster hemocytes treated with extracellular products from *A. crassostreae* experienced high mortality, indicating that extracellular proteins play a role in pathogenicity (10). One putative channel-forming hemolysin/cytolysin gene was identified. Additionally, BLASTX results reveal that the genome also contains 11 open reading frames with conserved regions associated with RTX toxins and related Ca^2+^ binding proteins, one with strong similarity to serralysin peptidase (11). An acyltransferase is located directly adjacent to two of these open reading frames (ORFs). These genes may be hemolysin/leukotoxin-type toxins affecting oyster hemocytes (12). Some of the annotated RTX toxins might also be involved in surface attachment (13). Additionally, a putative type IVA secretion system (T4ASS) was located. This T4ASS gene cluster is missing VirB1 and VirB7 in annotations from both RAST and IMG/ER. However, two ORFs within the cluster contain soluble lytic murein transglycosylase domains, which are the same class of proteins as VirB1 (14). Identification of VirB7 is often challenging since VirB7 has not been described in all T4SSs (14, 15). A partial T4BSS was also located and annotated; T4BSSs are often involved in pathogenesis (16).

### Nucleotide sequence accession numbers.

This whole-genome shotgun project has been deposited in DDBJ/ENA/GenBank under the accession no. LKBA00000000. The version described in this paper is version LKBA01000000.

## References

[1] Bricelj VM, Ford ES, Borrero FJ, Perkins FO, Rivara G, Hillman RE, Elston RA, Chang J 1992 Unexplained mortalities of hatchery-reared, juvenile oysters, *Crassostrea virginica* (Gmelin). J Shellfish Res 11:331–347.

[2] Davis CV, Barber BJ 1994 Size-dependent mortality in hatchery-reared populations of oysters, *Crassostrea virginica*, Gmelin 1791, affected by juvenile oyster disease. J Shellfish Res 13:137–142.

[3] Boettcher KJ, Geaghan KK, Maloy AP, Barber BJ 2005 *Roseovarius crassostreae* sp. nov., a member of the *Roseobacter* clade and the apparent cause of juvenile oyster disease (JOD) in cultured eastern oysters. Int J Syst Evol Microbiol 55:1531–1537. doi:10.1099/ijs.0.63620-0. 16014477

[4] Boettcher KJ, Barber BJ, Singer JT 1999 Use of antibacterial agents to elucidate the etiology of juvenile oyster disease (JOD) in *Crassostrea virginica* and numerical dominance of an α-proteobacterium in JOD-affected animals. Appl Environ Microbiol 65:2534–2539. 10347039 10.1128/aem.65.6.2534-2539.1999 PMC91374

[5] Karim M, Zhao W, Rowley D, Nelson D, Gomez-Chiarri M 2013 Probiotic strains for shellfish aquaculture: protection of eastern oyster, *Crassostrea virginica*, larvae and juveniles against bacterial challenge. J Shellfish Res 32:401–408. doi:10.2983/035.032.0220.

[6] Aziz RK, Bartels D, Best AA, DeJongh M, Disz T, Edwards RA, Formsma K, Gerdes S, Glass EM, Kubal M, Meyer F, Olsen GJ, Olson R, Osterman AL, Overbeek RA, McNeil LK, Paarmann D, Paczian T, Parrello B, Pusch GD, Reich C, Stevens R, Vassieva O, Vonstein V, Wilke A, Zagnitko O 2008 The RAST server: Rapid Annotations using Subsystems Technology. BMC Genomics 9:75. doi:10.1186/1471-2164-9-75. 18261238 PMC2265698

[7] Overbeek R, Olson R, Pusch GD, Olsen GJ, Davis JJ, Disz T, Edwards RA, Gerdes S, Parrello B, Shukla M, Vonstein V, Wattam AR, Xia F, Stevens R 2014 The SEED and the rapid annotation of microbial genomes using subsystems technology (RAST). Nucleic Acids Res 42:D206–D214. doi:10.1093/nar/gkt1226. 24293654 PMC3965101

[8] Brettin T, Davis JJ, Disz T, Edwards RA, Gerdes S, Olsen GJ, Olson R, Overbeek R, Parrello B, Pusch GD, Shukla M, Thomason JA III, Stevens R, Vonstein V, Wattam AR, Xia F 2015 RASTtk: a modular and extensible implementation of the RAST algorithm for building custom annotation pipelines and annotating batches of genomes. Sci Rep 5:8365. doi:10.1038/srep08365. 25666585 PMC4322359

[9] Markowitz VM, Chen IM, Palaniappan K, Chu K, Szeto E, Grechkin Y, Ratner A, Jacob B, Huang J, Williams P, Huntemann M, Anderson I, Mavromatis K, Ivanova NN, Kyrpides NC 2012 IMG: the integrated microbial genomes database and comparative analysis system. Nucleic Acids Res 40:D115–D122. doi:10.1093/nar/gkr1044. 22194640 PMC3245086

[10] Gómez-León J, Villamil L, Salger SA, Sallum RH, Remacha-Triviño A, Leavitt DF, Gómez-Chiarri M 2008 Survival of eastern oysters *Crassostrea virginica* from three lines following experimental challenge with bacterial pathogens. J Shellfish Res 79:95–105. doi:10.3354/dao01902. 18500026

[11] Altschul SF, Madden TL, Schäffer AA, Zhang J, Zhang Z, Miller W, Lipman DJ 1997 Gapped BLAST and PSI-BLAST: a new generation of protein database search programs. Nucleic Acids Res 25:3389–3402. doi:10.1093/nar/25.17.3389. 9254694 PMC146917

[12] Linhartová I, Bumba L, Mašín J, Basler M, Osička R, Kamanová J, Procházková K, Adkins I, Hejnová-Holubová J, Sadílková L, Morová J, Sebo P 2010 RTX proteins: a highly diverse family secreted by a common mechanism. FEMS Microbiol Rev 34:1076–1112. doi:10.1111/j.1574-6976.2010.00231.x. 20528947 PMC3034196

[13] Satchell KJ 2011 Structure and function of MARTX toxins and other large repetitive RTX proteins. Annu Rev Microbiol 65:71–90. doi:10.1146/annurev-micro-090110-102943. 21639783

[14] Gillespie JJ, Ammerman NC, Dreher-Lesnick SM, Rahman MS, Worley MJ, Setubal JC, Sobral BS, Azad AF 2009 An anomalous type IV secretion system in *Rickettsia* is evolutionarily conserved. PLoS One 4:e4833. doi:10.1371/journal.pone.0004833. 19279686 PMC2653234

[15] Cao TB, Saier MH Jr. 2001 Conjugal type IV macromolecular transfer systems of gram-negative bacteria: organismal distribution, structural constraints and evolutionary conclusions. Microbiology 147:3201–3214. doi:10.1099/00221287-147-12-3201. 11739753

[16] Nagai H, Kubori T 2011 Type IVB secretion systems of *Legionella* and other Gram-negative Bacteria. Front Microbiol 2:136. doi:10.3389/fmicb.2011.00136. 21743810 PMC3127085

